# Yield and costs of molecular diagnostics on thyroid cytology slides in the Netherlands, adapting the Bethesda classification

**DOI:** 10.1002/edm2.293

**Published:** 2021-09-02

**Authors:** Mehtap Derya Aydemirli, Marieke Snel, Tom van Wezel, Dina Ruano, Christianne M. H. Obbink, Wilbert B. van den Hout, Abbey Schepers, Hans Morreau

**Affiliations:** ^1^ Department of Pathology Leiden University Medical Center Leiden The Netherlands; ^2^ Department of Medical Oncology Leiden University Medical Center Leiden The Netherlands; ^3^ Division of Endocrinology Department of Internal Medicine Leiden University Medical Center Leiden The Netherlands; ^4^ Department of Surgery Haaglanden Medical Center The Hague The Netherlands; ^5^ Department of Biomedical Data Sciences Leiden University Medical Center Leiden The Netherlands; ^6^ Department of Surgery Leiden University Medical Center Leiden The Netherlands

**Keywords:** costs, FNAC, molecular diagnostics, next‐generation sequencing, thyroid cytology

## Abstract

**Objective:**

To evaluate our institutional experience with molecular diagnostics (MD) on thyroid cytology smears, evaluate the costs and describe MD guided clinical management of indeterminate Bethesda III/V thyroid nodules.

**Methods:**

We performed a retrospective review of 164 Bethesda III or V thyroid cytopathology reports subjected to MD from 2013 to 2020, that altered Bethesda classification or management. MD consisted of mutation and gene fusion analysis by next‐generation sequencing (NGS) of morphologically analysed and selected cytological slides. Findings were modelled to nationwide data on Bethesda incidences from ‘the Dutch Pathology Registry’ PALGA, and costs were estimated.

**Results:**

82 of 164 cases received an upgrade in Bethesda class. Twenty cases changed from Bethesda III to IV/V, 62 from Bethesda III or V to VI, and 72 remained unaltered. We estimate net savings with implementing MD, by preventing 454 repeat cytology and 326 (diagnostic) hemithyroidectomies, to be at least 2 million Euro annually in the Netherlands. Per Bethesda III and V patient, net savings would be about 100 Euro and 4100 Euro, respectively.

**Conclusion:**

NGS‐based MD on nucleic acids extracted directly from cytology slides is a feasible and cost saving tool for personalized management in indeterminate Bethesda III/V thyroid cytology. Based on the interpretation of our retrospective data, we assume that this approach results in less disease burden for the patient, reduced surgical interventions and complication risks, reduced sick leave, among others. Further evaluation of structural implementation of the presented approach in routine thyroid Bethesda III/V cytology in a prospective setting is warranted.

## Graphical abstract

We evaluated our institutional experience with molecular diagnostics (MD) on thyroid cytology smears, evaluated the costs and described MD guided clinical management of indeterminate Bethesda III/V thyroid nodules. NGS‐based MD on nucleic acids extracted directly from cytology slides is a feasible and cost saving tool for personalized management in indeterminate Bethesda III/V thyroid cytology.

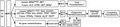

## 1 INTRODUCTION

Thyroid nodules have a high prevalence, up to 60% of the general population.^1^ However, only a minority (1.6–12%) of thyroid nodules is malignant.^2^, ^3^ Evaluation of thyroid nodules includes ultrasonography and fine‐needle aspiration cytology (FNAC). The cytology smears are classified into six Bethesda classes according to the Bethesda System for the Reporting of Thyroid Cytopathology (TBSRTC).^4^ Each Bethesda class implies a different risk of malignancy and subsequent recommended clinical management, along with other clinical considerations, although this may vary based on the available guidelines.^5^ Yet, as explicated in a recent review,^6^ the management of thyroid nodules with indeterminate cytology (Bethesda III to V) remains unclear, usually warranting diagnostic hemithyroidectomies or repeat FNACs. In case of atypia of undetermined significance or follicular lesion of unknown significance (AUS/FLUS, Bethesda III), FNAC is repeated (at least once) with the possibility of a different Bethesda outcome. According to the definitive histologic diagnosis obtained with a hemithyroidectomy, while taking into account other clinical considerations and current guideline recommendations, the subsequent management can be determined. In case of a benign lesion, no surgery might have been required; in case of malignancy, usually a second surgical procedure (completing thyroidectomy) is performed. As a consequence, many (in essence) unnecessary thyroid surgeries and repeat FNACs are performed due to indeterminate diagnosis.

Accrued insights in molecular tumorigenesis^7^ allow for the (auxiliary) implementation of molecular pathology in certain clinical cases and to help direct their subsequent management, as is also suggested in guidelines and literature.^1^, ^4^ Consequently, the extent of molecular diagnostics (MD) applied in thyroid cytology has expanded by the years. Single *BRAF^V600E^* detection with promising cost‐effectiveness studies^8^, ^9^ was expanded to cancer hotspot panels, to larger gene panels recently.^10^, ^11^, ^12^, ^13^, ^14^ In Bethesda III or V cytology cases in particular, MD may help direct a change in management. In contrast, MD would, with rare exceptions, not cause an alteration in management in case of Bethesda IV, in the setting of current (Dutch) guidelines. Molecular analysis of Bethesda IV cases may serve mainly to affirm a correct cytological classification of follicular lesions.

Several commercial molecular tests are available. These include for instance recently updated thyroid directed molecular diagnostics panels^15^, ^16^ ThyroSeq^®^ v3 Genomic Classifier (GC)^11^, ^12^, ^13^, ^14^ and Afirma^®^ Gene Sequencing Classifier (GSC).^17^, ^18^ Their application in clinical trials, along with cost‐effectiveness analyses,^19^ has also been studied. While promising, drawbacks of these commercial tests are their high costs^10^ and often the requirement of additional aspirates besides the regular FNAC; however, there are some recent rapports on the use of cytology slides also.^20^, ^21^

The application of molecular analysis to cytology slides is a suitable, affordable method and has shown promising results.^21^, ^22^, ^23^, ^24^, ^25^, ^26^, ^27^ During routine microscopic examination, representative areas of the regular cytology slides are marked by the pathologist. Then, nucleic acids for molecular testing can be isolated from these selected areas. Altogether, this technique may prove as a cheaper^28^ and a more feasible method in its routine implementation into conventional cytopathologic examination when compared to aforementioned commercial kits. Besides, the present approach may yield even more representative analysis results while requiring only very small amounts of nucleic acids from cells suspected for neoplasm that are carefully selected.^25^ In contrast, the use of commercial kits on additional cytological aspirates does not allow for microscopic examination or selection of the cells that are used for molecular analysis.

In this study, we evaluated our molecular diagnostics approach applied to microsections of routine thyroid FNAC slides selected during routine microscopic examination by the pathologist. To this end, we retrospectively reviewed our institutional cases with cytological indeterminate thyroid nodules graded as Bethesda III or Bethesda V, that were molecular diagnostically analysed, over seven years. The molecular diagnostics consist of targeted DNA and RNA next‐generation sequencing (NGS) using a custom AmpliSeq Cancer Hotspot Panel and a custom gene fusion FusionPlex panel. Crucial molecular alterations include DNA variants of genes such as *BRAF*, *NRAS*, *KRAS*, *HRAS*, *RET*, *PTEN*, *IDH1*, *GNAS*, *TP53*, *TERT*, *EIF1AX* and *DICER1*, and gene fusions involving *RET*, *NTRK*, *ALK*, *PPARG*, *THADA* and *BRAF*. Chromosomal copy number analysis is a recent update, not part of this retrospective study. Thereby, preoperative diagnostic indices may be obtained in nodules with indeterminate cytology (Bethesda III or V).

In addition, nationwide data were retrieved on Bethesda III to VI classifications of thyroid lesions. Reduced costs were estimated and modelled to national level. Ultimately, the presented method may serve as a feasible approach in both applicability (no additional material to routine cytology slides), cost‐effectiveness and patient safety by circumventing, in essence unnecessary, invasive procedures of repeat FNACs or diagnostic hemithyroidectomies.

### 1.1 Current evidence in the literature and interpretation of molecular diagnostic results

**FIGURE 1:**
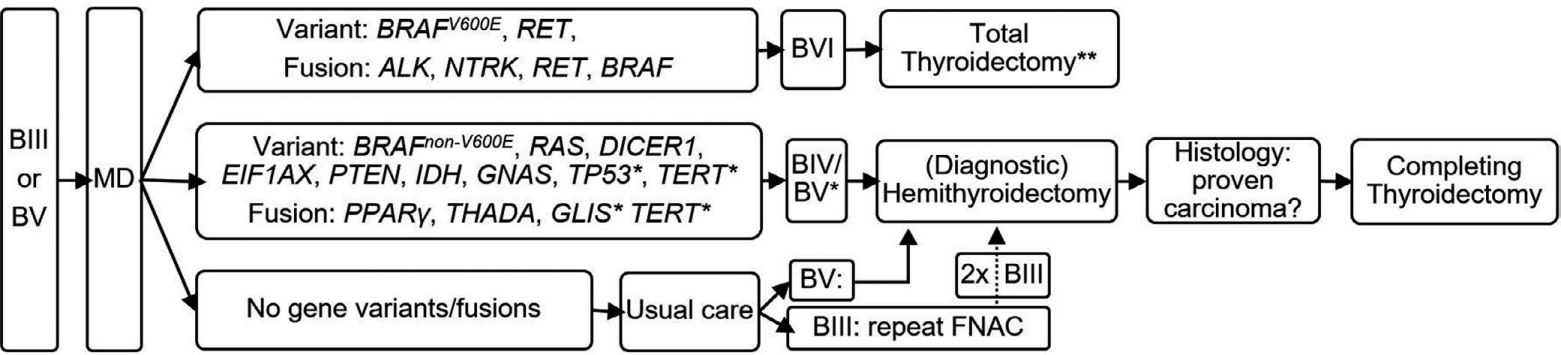
Proposed decision chart according to MD findings on thyroid cytopathology categorized as Bethesda III or V. The molecular alterations shown in the first box may modify the Bethesda grade to a Bethesda VI, followed by total thyroidectomy depending on diameter of the lesion. Please see main text for further details and clinical considerations. Please see main text for further details and clinical considerations. The molecular alterations shown the second box may, in order to acquire definite histopathological diagnosis, be followed by a (diagnostic) hemithyroidectomy. The molecular alterations shown the second box are mostly associated with follicular lesions and may occur in follicular adenoma/NIFTP/FVPTC/FTC; so would suit Bethesda IV. However, gene variants of *TP53*, *TERT* and *TERT* gene fusions (indicated with *) are not specific for a follicular lesion per se, and may be found in various types of thyroid lesions, and rather associated with a higher aggressive nature; so, on its own, may justify the modification a Bethesda III to a Bethesda V. *GLIS* gene fusions are associated with hyalinizing trabecular tumours and may modify the Bethesda class as Bethesda V. Molecular diagnostics include gene variant and gene fusion analysis of total nucleic acid using NGS with a custom Ampliseq Cancer Hotspot Panel and a custom Archer FusionPlex CTL Panel. N.B. *GLIS* and *TERT* gene fusions to be included in our updated custom panels, according to recent insights. **depending on diameter. BIII, Bethesda III; BV, Bethesda V; BVI, Bethesda VI; FNAC, fine‐needle aspiration cytology; MD, molecular diagnostics; NGS, next‐generation sequencing

With recent advancements in the understanding of pathophysiology and molecular mechanisms, panels for molecular testing, but also their clinical implications, keep evolving. For instance, detection of *BRAF* ^V600E^ gene variants^6^, ^29^ or gene fusions involving *ALK*,^29^, ^30^, ^31^, ^32^ *NTRK* ^33^, ^34^, ^35^ and *BRAF* ^31^ upgrades the Bethesda class to a Bethesda VI, as these are likely associated with papillary thyroid cancer (PTC) or may occur in poorly differentiated thyroid cancer (PDTC), anaplastic thyroid cancer (ATC) or, occasionally, medullary thyroid cancer (MTC), and malignancy should be expected in these cases, see Figure 1. The detection of (MTC‐associated) *RET* gene variants may upgrade the grade to Bethesda VI. *RET* fusions are likely associated with PTC or may occur in PDTC, ATC and MTC.^29^ As gene fusions occur more frequently in paediatric thyroid carcinoma than in the adult population, multiple studies have reported *RET*/PTC fusions in PTC,^36^ including the study of molecular diagnostics on thyroid nodules by Monaco et al reporting PTC for all detected *RET*/PTC fusions.^37^ Also in our experience, no *RET* fusions have been detected using NGS that were related to benign lesions so far (expert opinion, present study). Remarkably, as a diagnostic test *RET* fluorescence in situ hybridization is inferior due to substantial false‐positive results.^38^

In the cases harbouring the aforementioned gene alterations that suit Bethesda VI, if the lesion is evaluated as >1 cm, according to recommendations of current Dutch clinical guidelines, a total thyroidectomy may be considered. Thereby, two‐staged surgery in malignant Bethesda III and Bethesda V cases, including the exposure to risks for complications (including bleeding, wound infection) twice, but also additional costs, second hospitalization and recovery burden, may be circumvented with single‐staged surgery.

A (diagnostic) hemithyroidectomy may be considered (and modification of class to Bethesda IV, and/or occasionally (indicated with a *) Bethesda V), if variants *BRAF* ^non−V600E^,^29^, ^39^, ^40^ *RAS*,^29^, ^31^ *DICER1*,^41^, ^42^, ^43^ *EIF1AX*,^44^ *PTEN*,^45^, ^46^ *IDH1*,^47^ TP53*,^48^ *GNAS*, TERT*,^29^ or translocations of *PPARγ*,^31^, ^49^, ^50^ *THADA*,^31^, ^50^ TERT* are detected.^6^, ^32^ Indeed, these molecular alterations may yet occur in benign lesions, including non‐invasive follicular thyroid neoplasm with papillary‐like nuclear features (NIFTP)^50^ and follicular adenomas. However, the probable risk of malignancy, including (follicular variant of) PTC ((FV)PTC) or follicular thyroid carcinoma (FTC), requires definite histology. These molecular alterations, also see Figure 1, as being mostly associated with follicular lesions and may occur in follicular adenoma/NIFTP/FVPTC/FTC, would hence suit Bethesda IV. Some gene variants including *TP53*, *TERT* and *TERT* gene fusions (indicated with * in Figure 1) are rather not specific for a follicular lesion per se, and may be found in various types of thyroid lesion, so, on its own, may justify the modification of a Bethesda III to Bethesda V. *GLIS* gene fusions are associated with hyalinizing trabecular tumours and may modify the Bethesda class as Bethesda V. Concurrent *EIF1AX* and *RAS* variants are reported to occur in advanced thyroid carcinomas promoting tumorigenesis^51^; however, also NIFTP cases have been described harbouring concurrent *EIF1AX* and *RAS* variants.^44^, ^52^ *TERT* promoter variants, concurrent *TERT* and *RAS* variants or *TERT* and *BRAF* ^V600E^ gene variants are also reported to indicate a more aggressive nature with worse prognosis.^53^, ^54^, ^55^, ^56^, ^57^

In case of *GLIS* translocations (modification to Bethesda V), a lobectomy is the appropriate treatment, for this translocation is highly specific for hyalinizing trabecular tumour (a benign lesion).^58^, ^59^ Ultimately, according to histopathologic assessment of the diagnostic hemithyroidectomy, a completing thyroidectomy may be performed in case of proven carcinoma.

If no gene variants or fusions are detected using MD, usual care is followed. In case of Bethesda III, usual care includes surveillance for 2 years, with repeated ultrasound and FNAC after 6, 12 and 24 months; in case of a Bethesda III outcome for a second time, a diagnostic hemithyroidectomy is performed. In case of Bethesda V, usual care includes a diagnostic hemithyroidectomy. Regarding Bethesda IV lesions, currently detected molecular alterations may point to a follicular neoplasm, but are not indicative of the distinction between malignancy and benignity. Therefore, in Bethesda IV lesions, the likely appropriate approach would be a diagnostic hemithyroidectomy if both a genetic alteration may be detected or not. By default, all decisions are made while taking into account other clinical considerations and current guideline recommendations, as mentioned earlier.

## 2 MATERIALS AND METHODS

### 2.1 Case selection, retrospective review and data analysis

Nationwide data of anonymized reports encompassing Bethesda III to VI thyroid cytopathology over 7 years (01‐01‐2013 through 31‐12‐2019) including all thyroid cytopathology/surgery records per patient in PALGA: Dutch Pathology Registry were requested from and kindly provided by PALGA (The Nationwide Network and Registry of Histo‐ and Cytopathology in the Netherlands).^60^ The anonymized data set was handled in compliance with the Code of Conduct for the Use of Data in Health Research according to the Federation of Dutch Medical Scientific Societies (Federa), Codes of Conduct (https://www.federa.org/codes‐conduct). This study was approved by the Privacy Review Board of PALGA: Dutch Pathology Registry. According to the Central Committee on Research involving Human Subjects (CCMO), this type of study does not require approval from an ethics committee in the Netherlands. The present study was waived by the Medical Ethics Review Committee of the Leiden University Medical Center, Leiden (decision on 19 August 2020, registration number G20.104).

For an estimation of the incidence of FNACs classified as Bethesda III or V, the corresponding codes, as registered in PALGA for each report, were summed. This was performed both for nationwide and our institutional data.

Pathology reports of our institution regarding Bethesda III or V thyroid cytology with MD (before contingent initial thyroid surgery) at our institution were selected and retrospectively reviewed. Baseline characteristics, including sex, age and definitive histopathologic diagnosis, were retrieved. Data on MD outcome (likely pathogenic gene variants and gene fusions), definitive Bethesda class and clinical management (repeat FNAC, hemithyroidectomy, two‐staged total thyroidectomy, single‐staged total thyroidectomy, unknown) were collected. Crosstabulations were made of the different variables: MD outcome, year, Bethesda class III or V along with definitive (un)altered Bethesda class, clinical management, definitive histopathologic diagnosis, using IBM SPSS Statistics for Windows, version 25 (IBM Corp.).

### 2.2 Selection of thyroid cytopathologic cells and molecular diagnostics

MD was performed in the ISO15189 accredited Molecular Diagnostics Unit of the Pathology department of the LUMC (Leiden University Medical Center). The molecular analysis procedure of on nucleic acids isolated from cytology smears has been described previously.^61^ Briefly, cytologic smears are fixed in methanol and Giemsa stained. During routine microscopic examination by pathologists (HM), the area of interest, if discernible, on the inspected thyroid cytopathology smear classified as Bethesda III or V is precisely demarcated for tumour cell enrichment.

In separate tubes, slides are emerged in xylene until the coverslips detach. Slides are washed using ethanol washing steps (100%–70%–50%). After tissue rehydration, the demarcated cells are scraped off. Total nucleic acid was isolated using a fully automated DNA/RNA isolation system.^62^ In most cases, a single smear, yielding between 2 and 10 ng of nucleic acids, was sufficient for molecular analysis using NGS.

The molecular workup, gene variant analyses using a custom AmpliSeq™ Cancer Hotspot Panel (Thermo Fisher Scientific) and gene fusion analysis using Archer^®^ FusionPlex CTL panel (ArcherDX Inc.) (targeting 36 genes), has been described previously.^33^, ^41^, ^63^ Detected alterations classified as (likely) pathogenic (class 4 or 5, respectively) are reported.

Subsequently, MD results were interpreted by a registered molecular scientist in pathology (HM and TvW), and Bethesda class was regraded accordingly.

During the cause of the reviewed timeframe, the molecular workup was expanded. From 2013 through 2015, hotspot mutation analysis using Taqman BRAF allele‐specific hydrolysis assay was performed.^64^ From 2015, a custom AmpliSeq™ Cancer Hotspot Panel (CHP, Thermo Fisher Scientific) was used with frequent updates. Between 2015 and 2016, CHPv2 was used (targeting 50 genes); CHPv3 (targeting 60 genes) from 2016 through 2017; CHPv4 (targeting 74 genes) from 2017 through 2019; and CHPv6 (targeting 85 genes) in 2019. Archer^®^ FusionPlex CTL panel was implemented since 2017.

MD encompasses gene variants and gene fusions thus far known to be involved in thyroid tumorigenesis. These are discussed in the introduction section on literature and MD interpretation and the subsequent management strategy according to the decision chart in Figure 1. Crucial molecular alterations include DNA variants *BRAF*, *NRAS*, *KRAS*, *HRAS*, *RET*, *PTEN*, *IDH1*, *TP53*, TERT*; EIF1AX*, DICER1**, translocations of *RET*, *NTRK*, *ALK*, *PPARG*, *THADA*, *BRAF*, (GLIS***, TERT***) chromosomal copy number analysis; which are part of these assays. (*Among other genes, *TERT*, *EIF1AX* are additional target genes in the Cancer Hotspot Panel from v4 on, and **DICER1 in the Cancer Hotspot Panel v6. ***GLIS and *TERT* are additional fusion genes, along with *TERT* expression levels, in an updated custom ArcherDx CTL FusionPlex panel not yet implemented in this study.)

### 2.3 Estimations of reduced costs by implementing MD and modelling to nationwide data

Patient data from our institution regarding Bethesda III and V with molecular analysis were used for making estimations of reduced costs by implementing MD and subsequently modelled to the nationwide data. The proposed decision chart in Figure 1, based on the current practice at our institution, was used for modelling of saved costs. Additionally, a success rate for MD in Bethesda III and in Bethesda V cases, respectively, based on the institutional data, was adjusted for in the estimations.

Furthermore, as supplemental exploratory data, a separate assessment was made of additional reduction in costs, if follow‐up of Bethesda III cases with negative MD was to be surveillance (instead of usual care: repeat FNAC, and if indeterminate, followed by a diagnostic hemithyroidectomy).

For lack of individual data on healthcare utilization, we used a simple model to estimate Dutch societal costs with and without MD. The model only included medical cost of MD (€650 per patient), medical costs of FNAC and surgery (€600 and €6895), respectively, regardless of the type of surgery (diagnosis and treatment code for reimbursement (DBC code) 020112014 and 020112005, respectively, www.opendisdata.nl) and productivity costs (€200 per FNAC and €1600 per surgery). These productivity costs were estimated assuming 67% labour participation with on average 27 h per week (www.CBS.nl), with one day sick leave for FNAC and two weeks for surgery. Also, the share of female patients, age distribution and inflation was taken into account hereby.

## 3 RESULTS

### 3.1 Bethesda classification of thyroid nodules in the Netherlands

Estimations of Bethesda class incidences in the Netherlands, based on reported codes in PALGA from 2013 through 2019, were 7230 for Bethesda III and 2557 for Bethesda V (Table 1). For Bethesda III, the annual incidence increased through the years and ranged between 550 in 2013 and 1350 in 2019. For Bethesda V, the incidences ranged between 335 in 2013 and 436 in 2019.

**TABLE 1:** Estimated frequencies of Bethesda III or V classified thyroid cytopathology in the Netherlands

Frequencies (*n*)	2013–2019
Bethesda III	7230
Bethesda IV	3907
Bethesda V	2557
Bethesda VI	1946

### 3.2 Single institutional cases of Bethesda III/V thyroid cytopathology with MD

#### 3.2.1 Patient characteristics

In total, 164 FNAC cases with Bethesda III or V were analysed using MD at our institution from 2013 through 2019 (Table S1). 65 cases (40%) were classified as Bethesda III and 99 cases (60%) as Bethesda V. 74% (*n *= 122) of the patient cases was female, and the median age was 48 years (range 8–86). The histologic diagnoses were papillary thyroid carcinoma (PTC) in 64 cases (39%), follicular thyroid carcinoma (FTC) in seven cases (4%), Hürthle cell carcinoma (HCC) in a single case (1%), follicular variant of papillary thyroid carcinoma (FVPTC) in 15 cases (9%) and cribriform morular variant of papillary thyroid carcinoma (CMV‐PTC) in two cases (1%). Further, the histologic diagnosis was benign (without taking into account non‐invasive follicular thyroid neoplasm with papillary‐like nuclear features (NIFTP)) in 27 cases (17%), NIFTP in seven cases (4%) and in 41 cases (25%) there was no histology.

#### 3.2.2 Molecular diagnostics results

MD results from the MD analysis are listed in Table 2. In 54 cases (33%) a *BRAF^V600E^* gene variant and in 25 cases (15%) a *RAS* variant was detected; 9 (5%) cases had other gene variants. Gene fusions were detected in 10 (6%) cases. In 63 (38%) of all MD tested cases, no gene alterations were found. In seven cases (4% of total; six cases (9%) of Bethesda III and one case (1%) of Bethesda V), the quality of the material was insufficient for genetic variant testing.

**TABLE 2:** Gene variants or gene fusions detected using MD in 2013–2019

*n* (% within year)	2013–2019
No variant/fusion	63 (38)
*BRAF^V600E^*	54 (33)
*HRAS*	9 (5)
*NRAS*	14 (9)
*KRAS*	2 (1)
Other^a^	*9 (5)*
Fusion^b^	*10 (6)*
Unusable	7 (4)
Total	*168 (102)*

^a^ Other variants detected that are not listed in this table (all single cases): *PTEN*; *PTPN11*; *MUTYH* (concurrent with a *BRAF^V600E^* variant); *RET*; *TERTp* (which altered Bethesda class from III to V in a single case); *BRAF^non^* ^−^ *^V600E^*; *APC* splice variant; two cases with *PIK3CA* (of whom one concurrent with a *BRAF^V600E^* variant).

^b^ Gene fusion analysis was used from 2017 on. The 10 detected gene fusions involved the following partner genes: *THADA* (concurrent with a *BRAF^V600E^* variant); *PPARG* (two cases); *BRAF* (concurrent with a *BRAF^V600E^* variant); *RET* (five cases); *ALK*. Total numbers of detected, including concurrently occurring (shown in italics), gene alterations are shown. Unusable: insufficient quality of material for molecular analysis.

Gene fusion analysis was performed in 75 cases in total (46% of all cases with MD). Ten gene fusions were detected in 10 cases (13% of cases with (intended) fusion analysis), and no fusions were detected in 30 cases (40% of cases with (intended) fusion analysis); the quality of the material was insufficient for fusion analysis in 35 cases (47% of cases with (intended) fusion analysis).

As described earlier, the Bethesda classification could be altered based on the MD results. The frequencies of (un)altered Bethesda III or V cases upon MD are presented in Table 3. In retrospect, the Bethesda III that were upgraded upon MD to a Bethesda IV or V prevented repeat FNACs (20 of 65 cases, 31% within initial Bethesda III). Regarding Bethesda V cases that were reclassified as Bethesda IV upon MD, the appropriate management remained a diagnostic hemithyroidectomy (10 of 99 cases, 10% within initial Bethesda V). The Bethesda III cases that were reclassified as Bethesda VI prevented at least one repeat FNAC, an unnecessary diagnostic hemithyroidectomy and an unnecessary delay in treatment due to inappropriate surveillance in four cases (6% within initial Bethesda III). The shifts from a Bethesda V to a VI prevented an unnecessary diagnostic hemithyroidectomy in 58 cases (59% within initial Bethesda V).

**TABLE 3:** Frequencies of (un)altered Bethesda III/V upon MD in 2013–2019

*n* (% within initial Bethesda class)	2013–2019
Bethesda III →MD→ III	41 (63)
Bethesda III →MD→ IV	19 (29)
Bethesda III →MD→ V	1 (2)
Bethesda III →MD→ VI	4 (6)
Total initial Bethesda III cases	65 (100)
Bethesda V →MD→V	31 (31)
Bethesda V →MD→IV	10 (10)
Bethesda V →MD→VI	58 (59)
Total initial Bethesda V cases	99 (100)

Abbreviation: MD, molecular diagnostics.

The detected gene alterations with MD and the histopathologic diagnostic outcome are described in Table S2. Of the 41 (25% of all MD) Bethesda III FNACs without gene alterations or gene fusions, 25 cases did not undergo resection (61% of 41), 11 cases were benign (27% of 41), four cases were PTC (10% of 41) and a single case was FTC (2% of 41). However, the malignant ‘unaltered Bethesda III’ cases, concerned microcarcinomas of which no FNAC was obtained from in three of these five cases; instead the obtained FNAC correlated to co‐existent benign nodules in the thyroid. The other two of these five malignant ‘unaltered Bethesda III’ cases were found to harbour gene fusions on the resected material; however, fusion analysis had not been performed on the cytological material at the time. The Bethesda III/V classes that were altered and unaltered after MD and corresponding histopathologic diagnoses are shown in Table S3. Of the initially Bethesda III graded cases, 22% (14/65 cases) had a histologically proven carcinoma. Of the initially Bethesda V graded cases, 76% (75/99) had a histologically proven carcinoma.

### 3.3 Modelled data and costs in Bethesda III/V thyroid cytology

When taking together the 164 Bethesda III/V cases that were tested with MD (also see Table 3), we could model rough numbers to nationwide incidences of Bethesda III cases and Bethesda V cases in 2019.

About 1179 unnecessary surgical interventions are done annually in the Netherlands (743 in Bethesda III and 436 in Bethesda V).

Compared to usual care without MD, a strategy with the implementation of MD (also see Figure 1) reduces the number of diagnostic and completing hemithyroidectomies (see Table S4).

In Bethesda III patients, the average number of surgical procedures is reduced from 0.77 procedures without MD (ie a diagnostic hemithyroidectomy in 55%, followed by a completing hemithyroidectomy in 22%) to 0.72 procedures with MD (ie a total thyroidectomy in 6% and a diagnostic hemithyroidectomy in 31%, of which half is followed by a completing hemithyroidectomy; a 91% success rate for the MD test has been adjusted for). Thereby, about 77 surgical interventions in Bethesda III cases can be avoided annually in the Netherlands. Furthermore, without MD at least one repeat FNAC is done in all 1350 cases (100%), while no repeat FNAC would be required in 454 cases (34%) with MD. As a result, the average total costs are reduced from €7300 to €7200 per Bethesda III patient.

In Bethesda V patients, the number of surgical procedures is reduced because in the majority of patients, MD alters the classification to Bethesda VI, replacing separate diagnostic and completing hemithyroidectomies by one total thyroidectomy. Thus, the average number of surgical procedures is reduced from 1.84 to 1.26 per patient (a 99% success rate for the MD test has been adjusted for), and the average total costs are reduced from €15,500 to €11,350 per Bethesda V patient. Thereby, about 253 surgical interventions in Bethesda V cases can be avoided annually in the Netherlands.

For the annual 1350 Bethesda III and 436 Bethesda V patients in the Netherlands, about 326 unnecessary surgical interventions can be avoided. Total annual costs would decrease with 2 million Euro, from 17 million to 15 million Euro.

Furthermore, a separate exploratory assessment was made of additional reduction in costs if follow‐up of Bethesda III cases with negative MD was to be surveillance (instead of usual care with repeat FNAC, and if indeterminate, followed by a diagnostic hemithyroidectomy), see Table S4.

## 4 DISCUSSION

Many unnecessary surgical interventions for diagnostic means, over one thousand annually in the Netherlands, are performed due to indeterminate diagnostic classification of thyroid nodules. These include unnecessary diagnostic hemithyroidectomies in both benign and malignant cases. In the first case, this translates to a hemithyroidectomy that might not have been performed in retrospect. In the latter case, this translates to two‐staged surgical interventions, where a single‐staged total thyroidectomy could have been done, in retrospect. But also many repeat FNACs are done in case of cytology with atypia of undetermined significance/follicular lesion of unknown significance.

The use of MD on thyroid cytology slides graded Bethesda III or V may aid in directing the clinical management strategies. Thereby, about a third of unnecessary surgical interventions (ca 80 in Bethesda III cases and ca 250 in Bethesda V) and about a third of repeat FNAC can be avoided annually in the Netherlands. Moreover, the molecular diagnostics can be applied to morphologically selected areas on routine cytology slides, which is a very feasible method in its implementation, but also in costs. As mentioned before, molecular testing does currently not add much in Bethesda IV.

To test performance of MD, 164 cases of Bethesda III/V (*n *= 65 and 99, respectively) that had had MD at our institution, from 2013 through 2019, have retrospectively been analysed. In general, half of Bethesda III/V cases that were tested with MD received an upgrade in Bethesda class (*n *= 82, 50%) and, thereby, an indication for change in clinical management.

The Bethesda III cases that were altered to Bethesda IV/V (*n *= 20, 12%), required a hemithyroidectomy, instead of surveillance, ultrasonography (US) or repeat FNACs. The Bethesda III/V that were altered to Bethesda VI due to MD for proven malignancy (*n *= 62, 38%) had an indication for a total thyroidectomy. The management was not changed from usual care in the Bethesda III/V cases that remained unaltered after MD (*n *= 41, 25% and *n *= 31, 19%, respectively). The benign lesions (including NIFTP) on histopathologic outcome (total *n *= 34, 21%) corresponded to the unaltered Bethesda III and V cases (*n *= 11, 7% and *n *= 12, 7%, resp.) and Bethesda III and V that were altered to Bethesda IV after MD (*n *= 8, 5% and *n *= 3, 2%, resp.). The data of the implementation of MD to Bethesda III or V thyroid cytology from morphologically selected areas on the cytological slide in our institution from 2013 through 2019 were also modelled to nationwide Bethesda classifications. The estimated net saving, by preventing unnecessary diagnostic hemithyroidectomies and repeat FNACs, with the implementation of MD would be about 2 million Euro per year in the Netherlands. At the same time, it would result in less disease burden for the patient and reduced general surgery and complication risks.

Also, there are current tendencies towards de‐escalation of thyroid cancer surgery.^65^, ^66^ Patients with T1‐2N0M0 DTC lesions measuring <4 cm would not undergo a total thyroidectomy, but rather a hemithyroidectomy in case of Bethesda VI cytology (eg MD detected *BRAF^V600E^*). In that regard, the intended cost reductions intended to be achieved by implementing MD would be partially neutralized by conservative surgery a priori. However, also in those cases, MD could still provide information on tumour molecular characteristics suggestive of aggressiveness (eg concurrent *BRAF^V600E^* and *TERT* variants).

Moreover, for Bethesda III an interesting aspect of integrating molecular data with the morphology is that a wait‐and‐see approach can be advocated in cases with little clinical suspicion and negative molecular results, instead of a diagnostic hemithyroidectomy. That is something we are currently testing. Exploratory estimations for net saving are about 4.4 million Euro per year in the Netherlands with unnecessary hemithyroidectomies reduced to half. It must be stressed that in all cases, and especially here, clinical data such as sonographic or PET/CT information are important to take into account while evaluating individual cases. MD may be a valuable tool in helping direct management choice, and so are patient factors, such as a very young or old age, prior radiation exposure or family history.

(Commercial) molecular diagnostic tests include ThyroSeq and Afirma GEC (Veracyte Inc).^17^ In a recent clinical validation study, ThyroSeq V3 showed promising results with a sensitivity of 94% and specificity of 82%, and PPV and NPV were 66% and 97%, respectively, at a malignancy/NIFTP rate of 28%.^11^ Benign call rates for Afirma and ThyroSeq V3 were 54% and 61%, respectively, and specificity for Afirma is lower (68%) than for ThyroSeq V3.^12^ However, as molecular diagnostics are not differentiating between NIFTP and malignancy, taking these two entities together may lead to ambiguous interpretation of the stratifying strength of these molecular diagnostic tests. We prefer to include NIFTP in the Bethesda IV category for which hemithyroidectomy is appropriate, separately from malignant Bethesda VI for which total thyroidectomy may be chosen. Yet, in the face of de‐escalation, also in the latter case if <4 cm, a hemithyroidectomy may be chosen.

The costs of the commercial assays are high (ranging from about 3000 to 5000 USD).^19^ Nevertheless, despite these high costs, molecular testing was still more cost‐effective than diagnostic hemithyroidectomy with hypothetical modelling.^19^

Other studies also show the applicability and validity of molecular analyses to cytology smears, including thyroid cytology.^21^, ^22^, ^23^, ^24^, ^25^, ^26^, ^27^ The costs of MD as used in the present study on cytology smears are much lower (about 650 Euro). As in our study, material for NGS (as minimal as 2 ng of nucleic acids) is obtained from routine cytology smears upon dedicated morphological selection. Commercial kits mostly require additional aspirates for analysis, with no further morphological review of that material.

Limitations of this study include the retrospective nature of real‐world data analyses and modelled estimates of reduced costs. The detection range of genetic alterations by the panels used through time has expanded. Also, MD use is increasingly applied and panels were updated over the years. Consequently, the detected alterations may be an underrepresentation of actual alterations present. Furthermore, the proportion of unusable material for cDNA fusion analysis was much higher as compared to DNA gene variant analysis. Consequently, gene fusions, if present in those cases, may not have been picked up. Also, the number of cases in which MD was performed during the reviewed timeframe was limited due to exclusion of cases that were included in another ongoing study at the time (EfFECTS study, www.effects‐studie.nl). Nevertheless, the data give a good impression of overall advances that can be achieved by the routine implementation of MD in Bethesda III/V cases.

## 5 CONCLUSION

In this study, we used molecular profiling applied to cells selected from routine thyroid cytology smears graded as BIII or BV. Preoperative diagnostic indices in nodules with indeterminate cytology may ultimately serve as an auxiliary tool in directing towards a single‐, two‐staged thyroidectomy or diagnostic hemithyroidectomy. A more personalized treatment strategy can be followed using the MD guided diagnostic strategy. Thereby, patient safety is insured by circumventing invasive procedures, that is repeat FNAC or diagnostic hemithyroidectomy, if proven unnecessary by MD outcome. Also in its implementation on routinely obtained cytology smears without the need for separately obtained FNAC material, the presented method appeared to be feasible. Moreover, prevented (surgical) procedures showed cost‐effectiveness of the presented approach. The results of this study will pave the way for more research on its structural implementation in routine cytopathology in a prospective setting at larger scale.

## CONFLICT OF INTEREST

H.M.: advisor GenomeScan, Leiden, The Netherlands. The authors declare no conflict of interest that could be perceived as prejudicing the impartiality of this study.

## AUTHOR CONTRIBUTION

**Mehtap Derya Aydemirli:** Data curation (Lead); Formal analysis (Lead); Investigation (Lead); Visualization (Lead); Writing‐original draft (Lead); Writing‐review & editing (Lead). **Marieke Snel:** Formal analysis (Supporting); Resources (Equal); Writing‐review & editing (Equal). **Tom van Wezel:** Methodology (Equal); Resources (Equal); Software (Equal); Writing‐review & editing (Equal). **Dina Ruano:** Resources (Equal); Software (Lead). **Christianne M. H. Obbink:** Validation (Supporting). **Wilbert B. van den Hout:** Formal analysis (Supporting). **Abbey Schepers:** Resources (Equal); Validation (Supporting); Writing‐review & editing (Equal). **Hans Morreau:** Conceptualization (Lead); Methodology (Lead); Resources (Lead); Supervision (Lead); Writing‐original draft (Supporting); Writing‐review & editing (Lead).

## Supporting information

Supplementary Material

## Data Availability

The data that support the findings of this study are available in the supplementary material of this article.
