# Supplementary material for: Yield and costs of molecular diagnostics on thyroid cytology slides in the Netherlands, adapting the Bethesda classification
Source: Endocrinol Diabetes Metab. 2021 Sep 2;4(4):e00293. doi: 10.1002/edm2.293 (PMC8502216; doi:10.1002/edm2.293)
Supplement: Supplementary file 1 — Supplementary Material [file EDM2-4-e00293-s001.docx]

***Supplementary***

**Yield and costs of molecular diagnostics on thyroid cytology slides in the Netherlands; adapting the Bethesda classification**

Aydemirli MD^1,2^, Snel M^2^, van Wezel T^1^, Ruano D^1^, Obbink CMH^3^, van den Hout WB^4^, Schepers A^3^, Morreau H^1^*

*Corresponding author: Prof. dr. Hans Morreau, J.Morreau@lumc.nl

**Supplementary Table 1** Characteristics of 164 Bethesda III/V patient cases subjected to MD (2013 – 2019)

| Characteristics | Total *n*=164** |
| --- | --- |
| Female, *n* (%) | 122 (74) |
| Age, median (range) | 48 (8–86) |
| Histology, *n* (%) |  |
| No histology | 41 (25) |
| PTC | 64 (39) |
| FTC | 7 (4) |
| HCC | 1 (1) |
| FVPTC | 15 (9) |
| CMV-PTC | 2 (1) |
| Benign* | 27 (17) |
| NIFTP | 7 (4) |
| Bethesda III/V, *n* (%) |  |
| Bethesda III | 65 (40) |
| Bethesda V | 99 (60) |

*NIFTP is shown separately from ‘Benign’

**Of note, regarding the number of selected reports and Leiden University Medical Center (LUMC) cases for review: the actual number of patients is 163, however, one case had had two separate FNACs on two different occasions in contralateral thyroid lobes with different outcome. Whereas the left lobe harbored a *NRAS* variant in a FVPTC (Bethesda III to IV), the right lobe harbored a *HRAS* variant in a follicular adenoma (Bethesda V to IV) and the treatment was a single-staged thyroidectomy. Hence, it was decided to register the two different FNACs as separate cases (hence a total of 164 cases) in this one patient.

PTC, papillary thyroid carcinoma; FTC, follicular thyroid carcinoma; HCC, Hürthle cell carcinoma; FV-PTC, follicular variant of papillary thyroid carcinoma; CMV-PTC, cribriform morular variant of papillary thyroid carcinoma; NIFTP, non-invasive follicular thyroid neoplasm with papillary-like nuclear features.

**Supplementary Table 2** Histopathologic diagnostic outcome and MD outcome (2013 – 2019)

| *n* (% within column) | None | *BRAF^V600E^* | *HRAS* | *NRAS* | *KRAS* | Other* | Fusion** | Unusable | Total |
| --- | --- | --- | --- | --- | --- | --- | --- | --- | --- |
| No histology | 22 (35) | 7 (13) | 2 (22) | 2 (14) | 1 (50) | 2 (29) | 1 (12.5) | 4 (57) | 41 (25) |
| PTC | 11 (18) | 46 (85) | 0 | 2 (14) | 0 | 0 | 4 (50) | 1 (14) | 64 (39) |
| FTC | 4 (6) | 0 | 2 (22) | 0 | 0 | 0 | 1 (12.5) | 0 | 7 (4) |
| HCC | 1 (2) | 0 | 0 | 0 | 0 | 0 | 0 | 0 | 1 (1) |
| FVPTC | 4 (6) | 1 (2) | 2 (22) | 3 (21) | 1 (50) | 2 (29) | 2 (25) | 0 | 15 (9) |
| CMV-PTC | 1 (1) | 0 | 0 | 0 | 0 | 1 (14) | 0 | 0 | 2 (1) |
| Benign*** | 20 (32) | 0 | 1 (11) | 2 (14) | 0 | 2 (29) | 0 | 2 (29) | 27 (17) |
| NIFTP | 0 | 0 | 2 (22) | 5 (36) | 0 | 0 | 0 | 0 | 7 (4) |
| Total | 63 (100) | 54 (100) | 9 (100) | 14 (100) | 2 (100) | 7 (100) | 8 (100) | 7 (100) | 164 (100) |

*Other variants detected that are not listed in this table (all single cases): *PTEN*; *PTPN11*; *MUTYH* (concurrent with a *BRAF^V600E^* variant); *RET*; *TERTp*; *BRAF^non-V600E^*; *APC* splice variant; two cases with *PIK3CA* (of whom one concurrent with a *BRAF^V600E^* variant); concurrently occurring variants have been omitted from the table.

**Gene fusions detected involving the following partner genes: *THADA* (concurrent with a *BRAF^V600E^* variant); *PPARG* (2 cases); *BRAF* (concurrent with a *BRAF^V600E^* variant); *RET* (5 cases); *ALK*; the concurrently occurring fusions have been omitted from the table.

***NIFTP is shown separately from ‘Benign’.

Unusable: insufficient quality of material for molecular analysis. PTC, papillary thyroid carcinoma; FTC, follicular thyroid carcinoma; HCC, Hürthle cell carcinoma; FV-PTC, follicular variant of papillary thyroid carcinoma; CMV-PTC, cribriform morular variant of papillary thyroid carcinoma; NIFTP, non-invasive follicular thyroid neoplasm with papillary-like nuclear features.

All *BRAF^V600E^* variants with a histologic outcome (n=47, 100%) were PTC (n=46) or FVPTC (n=1); 7 cases harboring *BRAF^V600E^* variants did not undergo resection yet.

*RAS* variants were detected in 25 cases (15% of all MD); these correlated to benign lesions in 10 cases (50% of 20 histologically confirmed cases), to FVPTC in 6 cases (30% of 20), PTC in 2 cases (10% of 20); 5 cases did not undergo resection yet (20% of all 25 detected *RAS* cases).

Regarding 9 (5% of all MD) other variants detected in FNAC material and corresponding histopathologic outcome, these are as follows (all single cases): *PTEN* and follicular adenoma; *PTPN11* (pathogenicity unknown) and lymphocytic thyroiditis; *MUTYH* (concurrent with a *BRAF^V600E^* variant) and no histology; *RET* and no histology; *TERTp* and FVPTC; *BRAF^non-V600E^* and FVPTC; *PIK3CA* and no histology; *PIK3CA* (concurrent with *BRAF^V600E^*) and PTC; *APC* splice variant and CMV-PTC. *Of note, the two concurrently occurring variants have been omitted from the table and dataset for other analyses.*

Ten (6% of all MD) gene fusions were detected involving the following fusion partner genes and histopathologic outcome (all single cases, unless specified otherwise): *THADA* (concurrent with a *BRAF^V600E^* variant) and PTC; *PPARG* (2 cases) and FTC and FVPTC, respectively; *BRAF* (concurrent with a *BRAF^V600E^* variant) and PTC; *RET* (5 cases) and PTC in 3 cases, FVPTC in a single case, no histology in a single case; *ALK* and PTC. *Of note, the two concurrently occurring fusions have been omitted from the table and dataset for other analyses.*

Of seven (4% of all MD) cases with unusable FNAC material for MD, 4 cases did not undergo resection, 2 cases were benign and 1 case had a PTC.

**Supplementary Table 3** (Un)altered Bethesda III/V and histopathologic diagnostic outcome (2013 – 2019)

| *n* (% within row) | No histology | PTC | FTC | HCC | FVPTC | CMV-PTC | Benign* | NIFTP | Total |
| --- | --- | --- | --- | --- | --- | --- | --- | --- | --- |
| Bethesda III 🡪MD🡪 III | 25 (61) | 4 (10) | 1 (2) | 0 | 0 | 0 | 11 (27) | 0 | 41 (100) |
| Bethesda III 🡪MD🡪 IV | 6 (32) | 2 (10.5) | 1 (5) | 0 | 2 (10.5) | 0 | 3 (16) | 5 (26) | 19 (100) |
| Bethesda III 🡪MD🡪 V | 0 | 0 | 0 | 0 | 1 (100) | 0 | 0 | 0 | 1 (100) |
| Bethesda III 🡪MD🡪 VI | 1 (25) | 3 (75) | 0 | 0 | 0 | 0 | 0 | 0 | 4 (100) |
| Bethesda V 🡪MD🡪 V | 1 (3) | 8 (26) | 3 (10) | 1 (3) | 4 (13) | 2 (6) | 12 (39) | 0 | 31 (100) |
| Bethesda V 🡪MD🡪 IV | 0 | 0 | 2 (20) | 0 | 5 (50) | 0 | 1 (10) | 2 (20) | 10 (100) |
| Bethesda V 🡪MD🡪 VI | 8 (14) | 47 (81) | 0 | 0 | 3 (5) | 0 | 0 | 0 | 58 (100) |
| Total | 41 (25) | 64 (39) | 7 (4) | 1 (1) | 15 (9) | 2 (1) | 27 (17) | 7 (4) | 164 (100) |

*NIFTP is shown separately from ‘Benign’

MD, molecular diagnostics; PTC, papillary thyroid carcinoma; FTC, follicular thyroid carcinoma; HCC, Hürthle cell carcinoma; FV-PTC, follicular variant of papillary thyroid carcinoma; CMV-PTC, cribriform morular variant of papillary thyroid carcinoma; NIFTP, non-invasive follicular thyroid neoplasm with papillary-like nuclear features.

Unaltered Bethesda III cases (41 in total, 25% of all MD) had no histology in 25 cases (61% of 41), benign lesions in 11 cases (27% of 41), PTC in 4 cases (10% of 41) and FTC in a single case (2% of 41). However, the malignant ‘unaltered Bethesda III’ cases, concerned microcarcinomas of which no FNAC was obtained from in 3 of these 5 cases; instead the obtained FNAC correlated to co-existent benign nodules in the thyroid. The other 2 of these 5 malignant ‘unaltered Bethesda III’ cases, were found to harbor gene fusions on the resected material, however, gene fusion analysis had not been performed on the cytological material. The Bethesda III to a IV cases (19 in total, 12%) had no histology in 6 cases (32% of 19), and of the histology-proven cases benign lesions in 8 cases (including NIFTP) (62% of 13), PTC in 2 cases (15% of 13), FVPTC in 2 cases (15% of 13), FTC in a single case (8% of 13). One Bethesda III to a V case concerned a FVPTC (1% of all MD). Bethesda III to VI cases (4 in total, 2% of all MD) had no histology in one case (25% of 4), and PTC in 3 cases (100% of all 3 histology-proven cases).

Unaltered Bethesda V cases (31 in total, 19% of all MD) had no histology in one case (3% of 31), of the histology-proven cases benign lesions in 12 cases (40% of 30), PTC in 8 cases (27% of 30), FVPTC in 4 cases (13% of 30), CMV-PTC in a two cases (7% of 30), FTC in 3 cases (10% of 30) and HCC in a single case (3% of 30). Bethesda V to IV cases (10 in total, 6% of all MD) concerned benign lesions in 3 cases (including NIFTP) (30% of 10), FTC in 2 cases (20% of 10), FVPTC in 5 cases (50% of 10). Bethesda V to VI cases (58 in total, 35% of all MD) concerned PTC in 47 cases (94% of the 50 histology-proven cases), FVPTC in 3 cases (6% of 50 histology-proven cases) and lacked histology in 8 cases (14% of 58).

**Supplementary Table 4**: Average costs per patient (in €) for care with and without MD, in Bethesda III and Bethesda V patients

| **MD care for Bethesda III patients (n=1350)** | | | | | | **Usual care** without MD **for Bethesda III patients (n=1350)** | | | | |
| --- | --- | --- | --- | --- | --- | --- | --- | --- | --- | --- |
|  |  | **Strategy change by positive MD** | | ***Exploratory*:**  **Surveillance for negative MD in BIII** | |  |  |  |  | |
| **Provided care** | **Test results** | **n** | **Costs** | **n** | **Costs** | **Provided care** | **Test results** | **n** | **Costs** | |
| MD, Re-FNAC  MD, Re-FNAC, dHT  MD, Re-FNAC, dHT, cHT  MD only  MD, dHT  MD, dHT, cHT  MD, dHR, cHT  MD, TT  **Total** | Unusable  Unusable  Unusable  63% BIII  29% BIV, 50% dHT-  29% BIV, 50% dHT+  2% BV  6% BVI | 608  261  27  0  180  180  19  76  **1350** | 1,400  9,900  18,300  650  9,100  17,500  17,500  9,100  **7,200** | 55  40  27  775  180  180  19  76  **1350** | 1,400  9,900  18,300  650  9,100  17,500  17,500  9,100  **5,400** | Re-FNAC  Re-FNAC, dHT  Re-FNAC, dHT, cHT  **Total** | 45% BII  55% BIII+, 60% dHT-  55% BIII+, 40% dHT+ | 608  446  297  **1350** | 760  9,200  17,700  **7,300** | |
| **MD care for Bethesda V patients (n=436)** | | | | | | **Usual care** without MD **for Bethesda V patients (n=436)** | | | | |
| **Provided care** | **Test results** | **n** | **Costs** |  |  | **Provided care** | **Test results** | **n** | **Costs** | |
| MD, dHT  MD, dHT, cHT  MD, dHT  MD, dHT, cHT  MD, dHT  MD, dHT, cHT  MD, TT  **Total** | Unusable  Unusable  10% BIV, 30% dHT-  10% BIV, 70% dHT+  31% BV, 40% dHT-  31% BV, 60% dHT+  59% BVI | 1  4  13  31  54  81  253  **436** | 9,100  17,500  9,100  17,500  9,100  17,500  9,100  **11,350** |  |  | dHT  dHT, cHT  dHT  dHT, cHT  dHT, cHT  **Total** | 30% dHT-  70% dHT+  40% dHT-  60% dHT+  100% dHT+ | 13  31  55  82  255  **436** | 8,400  16,800  8,400  16,800  16,800  **15,500** |  |

A success rate of 91% for the MD in Bethesda III cases and 99% in Bethesda V cases has been corrected for in the calculations. Regarding the use of MD in Bethesda III, ‘strategy change by positive MD’ is according to current standard of care (as also presented in Figure 1). An additional separate column (grey color) has been added for exploratory estimations if negative MD results in Bethesda III cases were also to be used to stratify for surveillance as follow-up management strategy.

MD, molecular diagnostics; dHT, diagnostic hemithyroidectomy; cHT, completing hemithyroidectomy; TT, total thyroidectomy;
Re-FNAC, repeat fine needle aspiration cytology; n, annual number of patients in the Netherlands; Unusable, insufficient quality of material for MD.

*Exploratory estimations for expectant management for MD negative Bethesda III*

A strategy based on positive MD and in addition expectant management in case of Bethesda III patients with negative MD, would additionally reduce costs. The latter strategy is not yet implemented in standard care. In Bethesda III patients, the average number of surgical procedures would be reduced from 0.77 procedures without MD (i.e. a diagnostic hemithyroidectomy in 55%, followed by a completing hemithyroidectomy in 22%) to 0.55 procedures with MD (i.e. a total thyroidectomy in 6% and a diagnostic hemithyroidectomy in 31%, of which half is followed by a completing hemithyroidectomy; a 91% success rate for the MD test has been taken into account). Thereby, about 298 surgical interventions in Bethesda III cases can be avoided annually in the Netherlands. For the annual 1350 Bethesda III and 436 Bethesda V patients in the Netherlands, about 551 unnecessary surgical interventions can be avoided. Furthermore, without MD at least one repeat FNAC is done in all 1350 cases (100%), while no repeat FNAC would be required in 1229 cases (91%) with MD. As a result, the average total costs would be reduced from €7,300 to €5,400 per Bethesda III patient. So, in case of expectant management for Bethesda III cases that tested negative for MD, in addition to MD in Bethesda V as calculated above, total annual costs would decrease from 17 million to 12.6 million Euro. Thus, total savings would be 4.4 million Euro.
